# Activation of cGMP/Protein Kinase G Pathway in Postconditioned Myocardium Depends on Reduced Oxidative Stress and Preserved Endothelial Nitric Oxide Synthase Coupling

**DOI:** 10.1161/JAHA.112.005975

**Published:** 2013-02-22

**Authors:** Javier Inserte, Victor Hernando, Úrsula Vilardosa, Elena Abad, Marcos Poncelas‐Nozal, David Garcia‐Dorado

**Affiliations:** 1 Laboratory of Experimental Cardiology, Vall d'Hebron University Hospital and Research Institute, Universitat Autònoma de Barcelona, Barcelona, Spain (J.I., V.H., V., E.A., M.P.N., D.G.D.)

**Keywords:** cardioprotection, nitric oxide synthase, oxidative stress, postconditioning, reperfusion injury

## Abstract

**Background:**

The cGMP/protein kinase G (PKG) pathway is involved in the cardioprotective effects of postconditioning (PoCo). Although PKG signaling in PoCo has been proposed to depend on the activation of the phosphatidylinositol 3‐kinase (PI3K)/Akt cascade, recent data bring into question a causal role of reperfusion injury signaling kinase (RISK) in PoCo protection. We hypothesized that PoCo increases PKG activity by reducing oxidative stress–induced endothelial nitric oxide synthase (NOS) uncoupling at the onset of reperfusion.

**Methods and Results:**

Isolated rat hearts were submitted to 40 minutes of ischemia and reperfusion with and without a PoCo protocol. PoCo reduced infarct size by 48% and cGMP depletion. Blockade of cGMP synthesis (1*H*‐[1,2,4]oxadiazolo[4,3‐**a**]quinoxalin‐1‐one) and inhibition of PKG (KT5823) or NOS (l‐NAME) abolished protection, but inhibition of PI3K/Akt cascade (LY294002) did not (n=5 to 7 per group). Phosphorylation of the RISK pathway was higher in PoCo hearts. However, this difference is due to increased cell death in control hearts because in hearts reperfused with the contractile inhibitor blebbistatin, a drug effective in preventing cell death at the onset of reperfusion, RISK phosphorylation increased during reperfusion without differences between control and PoCo groups. In these hearts, PoCo reduced the production of superoxide (O_2_^−^) and protein nitrotyrosylation and increased nitrate/nitrite levels in parallel with a significant decrease in the oxidation of tetrahydrobiopterin (BH_4_) and in the monomeric form of endothelial NOS.

**Conclusions:**

These results demonstrate that PoCo activates the cGMP/PKG pathway via a mechanism independent of the PI3K/Akt cascade and dependent on the reduction of O_2_^−^ production at the onset of reperfusion, resulting in attenuated oxidation of BH_4_ and reduced NOS uncoupling.

## Introduction

Ischemic postconditioning (PoCo), a cardioprotective strategy consisting of brief episodes of intermittent coronary reocclusions applied at the onset of reperfusion, represents a feasible approach to limit infarct size in the clinical setting. An increasing number of studies suggest that PoCo enhances myocardial salvage and improves clinical outcomes in patients with acute myocardial infarction who are receiving primary percutaneous coronary intervention,^1–2^ but its exact mechanisms have not been completely elucidated.^3^

Experimental evidence demonstrates that delayed normalization of intracellular pH during initial reperfusion plays a determinant role in the effectiveness of a PoCo protocol.^4–5^ In addition, the use of nitric oxide (NO) synthase (NOS), guanylyl cyclase (GC), or protein kinase G (PKG) inhibitors abolished the infarct‐sparing effect of PoCo in different studies,^6–8^ confirming the involvement of the NO‐dependent activation of the cGMP/PKG pathway in the cardioprotection induced by PoCo. Among other mechanisms, it was recently proposed that PKG activation contributes to PoCo protection by delaying normalization of pHi during reperfusion, probably via PKG‐dependent inhibition of Na+/H+‐exchanger.^7^ Activation of the cGMP/PKG pathway by PoCo has been largely proposed to be dependent on the activation of the phosphatidylinositol 3‐kinase (PI3K)/Akt cascade.^3^ However, a recent study in the in situ pig model suggests that increased reperfusion injury signaling kinase (RISK) phosphorylation is not associated with a reduction in infarct size, questioning a causal role of RISK in the protection induced by PoCo.^9^

On the other hand, it is well accepted that reactive oxygen species (ROS), including superoxide (O_2_^−^), are acutely formed and released during the first minutes of reperfusion.^10–11^ Formation of O_2_^−^ has been proposed to reduce the bioavailability of NO by increasing the production of peroxynitrite (ONOO^−^) and the oxidation of tetrahydrobiopterin (BH_4_), a cofactor needed for NOS coupling that has been shown to be largely depleted during ischemia–reperfusion.^12^ PoCo, by preventing an abrupt reperfusion, may reduce the burst of ROS and increase the NO‐mediated activation of the cGMP/PKG pathway. However, no studies have been performed to investigate the effect of PoCo on NOS coupling. The aims of our study were to (1) demonstrate that activation of PKG/cGMP pathway is independent of PI3K/Akt signaling and that the increased phosphorylation of RISK observed in PoCo is a consequence but not a cause of the reduction in cell death in a rodent model and (2) determine whether PoCo activates the cGMP/PKG pathway by increasing the coupling of eNOS as consequence of reduced oxidative stress and preserved levels of BH_4_.

## Methods

The experimental procedures conformed to the “Guide for the Care and Use of Laboratory Animals” published by the US National Institutes of Health (NIH Publication No. 85‐23, revised 1996) and were approved by the Research Commission on Ethics of the Hospital Vall d'Hebron.

### Isolated Perfused Rat Heart Preparation

Male Sprague–Dawley rats (300 to 350 g) were anesthetized with sodium pentobarbital (100 mg/kg). Hearts were removed, mounted onto a Langendorff apparatus, and perfused with a modified Krebs–Henseleit bicarbonate buffer (KHB, in mmol/L: NaCl 140, NaHCO_3_ 24, KCl 2.7, KH_2_PO_4_ 0.4, MgSO_4_ 1, CaCl_2_ 1.8, and glucose 11) equilibrated with 95% O_2_ and 5% CO_2_ as described previously.^5^ Flow rate was initially adjusted to produce a perfusion pressure of 60 mm Hg and was held constant thereafter. Left ventricular (LV) pressure was monitored through the use of a water‐filled latex balloon inserted into the left ventricle and inflated to obtain an end‐diastolic pressure (LVEDP) between 6 and 8 mm Hg. LV developed pressure was calculated as the difference between LV systolic pressure and LVEDP. Perfusion pressure was continuously recorded using a pressure transducer connected to the perfusion line.

### Experimental Protocol

**Figure 1.**
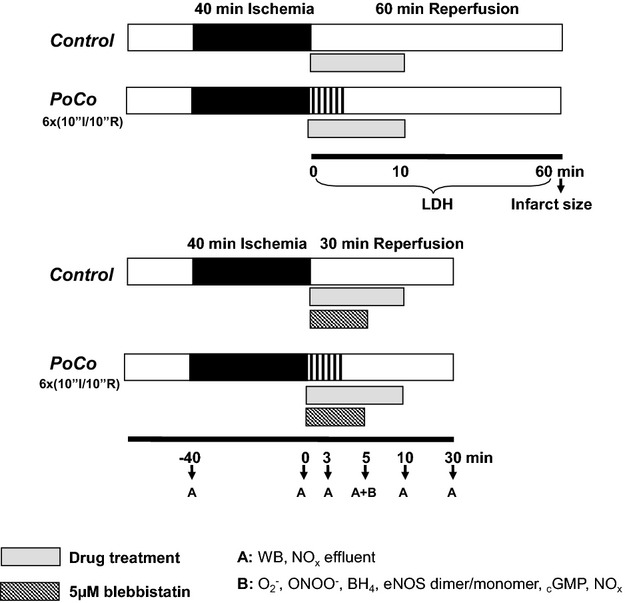
Experimental protocol. In a first set of experiments, isolated rat hearts were subjected to 40 minutes of ischemia and 60 minutes of reperfusion with and without postconditioning (PoCo) consisting of 6 cycles of 10 seconds of ischemia and 10 seconds of reperfusion. In a second set of experiments, control and PoCo hearts received the contractile inhibitor blebbistatin at the onset of reperfusion. Samples for Western blots and measurement of NO_x_ released to the coronary effluent were obtained at different times (A) and other parameters were measured after 5 minutes of reperfusion (B). In both set of experiments, the different drugs were given during the first 10 minutes of reperfusion (drug treatment: KT‐5823, ODQ, l‐NAME, LY‐294002, SIN‐1, BH_4_). LDH indicates lactate dehydrogenase; NO_x_, total nitrate/nitrite; eNOS, endothelial nitric oxide synthase; WB, Western blots; ONOO^−^, peroxynitrite; ODQ, 1*H*‐[1,2,4]oxadiazolo[4,3‐**a**]quinoxalin‐1‐one; BH_4_, tetrahydrobiopterin; O_2_^−^, superoxide; SIN‐1, 3‐morpholinosydnonimine.

The different experimental protocols are illustrated in Figure 1. In control group, hearts were subjected to global ischemia for 40 minutes followed by reperfusion of a different duration. PoCo was achieved with a previously established protocol consisting of 6 cycles of 10‐second reperfusion–10‐second occlusion (PoCo group).^5^ In additional groups of hearts, the inhibitor of soluble guanylate cyclase (3 μmol/L 1*H*‐[1,2,4]oxadiazolo[4,3‐**a**]quinoxalin‐1‐one [ODQ], Sigma‐Aldrich), PKG (1 μmol/L KT‐5823, Sigma‐Aldrich), NOS (100 μmol/L l‐NAME Sigma‐Aldrich), or PI3K (15 μmol/L LY294002, Sigma‐Aldrich) was added during the first 10 minutes of reperfusion.

In those experiments aimed to analyze a variable different than cell death, the selective contractile inhibitor blebbistatin (10 μmol/L, Merck)^13^ was added during reperfusion or after 60 minutes in hearts perfused with oxygenated buffer (Nx). Blebbistatin prevents sarcolemmal rupture and cell death by reducing the mechanical stress caused by the excessive contractile activation occurring at the onset of reperfusion. It was used to discard the possibility that any variation on the measured parameters was a mere consequence of differences in cell death associated with sarcolemmal disruption. This strategy has been previously used with the less‐specific contractile inhibitor butanedione monoxime.^14^

### Quantification of Cell Death

Lactate dehydrogenase (LDH) activity was spectrophotometrically measured in the coronary effluent throughout the reperfusion period. After 60 minutes of reperfusion, heart slices were incubated in 1% TTC to outline the area of necrosis as previously described (n=7 per group except controls with l‐NAME, ODQ, and KT‐5823, n=5).^15^ Previous studies demonstrated that in the Langendorff rat model, reperfusion for 60 minutes is sufficient for acute assessment of infarct size.^16–17^

### Protein Phosphorylation

Protein phosphorylation was measured in heart samples homogenized in buffer containing Tris‐HCl 50 mmol/L, NaCl 150 mmol/L, EDTA 10 mmol/L, DTT 1 mmol/L, NaF 10 mmol/L, Na_3_VO_4_ 2 mmol/L, 1% Triton X‐100, and 1% Protease Inhibitor Cocktail (Sigma), pH 7.3, and centrifugated at 15 000*g* during 15 minutes. Samples from supernatants were separated via electrophoresis on 7.5% to 12% SDS gels with a Tris‐glycine‐SDS running buffer (Sigma), transferred onto nitrocellulose membrane (Hybond ECL, Amersham), blocked with 5% PhosphoBlocker Blocking Reagent (Cell Biolabs, Inc), and immunoblotted with antibodies against eNOS Ser1177 (BD Transduction Laboratories), Akt Ser473, GSK‐3β Ser9, and ERK1/2 Thr202/Tyr204 (Cell Signaling Technology). Membranes were reprobed with antibodies against the total form of the respective protein. Bands were detected via chemiluminiscence (SuperSignal West Dura Extended Duration Substrate, Pierce) and quantified using a charge‐coupled device system (Image Reader LAS‐3000, Fujifilm) and image analysis software (Image Gauge, Fujifilm). Hearts normoxically perfused (Nx) for 60 minutes were used as reference of the stress activation associated with the Langendorff preparation.^18^ Equal protein load was confirmed with Ponceau staining. Signals from the phosphorylated form of a given kinase were normalized to its total content and final values were obtained from 4 different samples.

### Quantification of Superoxide Production

Oxidation of the cell permeant fluorescent dye dihydroethidium (DHE) to ethidium and hydroxyethidium was used to measure O_2_^−^.^12^ Hearts were perfused with 8 μmol/L DHE and blebbistatin during the first 5 minutes of reperfusion and homogenated in buffer containing Tris‐HCl 50 mmol/L, NaCl 150 mmol/L, EDTA 10 mmol/L, 1% Triton X‐100, and 1% Protease Inhibitor Cocktail (Sigma), pH 7.3 (n=6 per group except for PoCo groups reperfused with ODQ and l‐NAME [n=5 per group]). Two hundred microliters of each sample was read in a Spectra MAX GEMINI plate reader (510 nm excitation and 590 nm emission) to measure the formation of the O_2_^−^‐derived ethidium and hydroxyethidium. Background fluorescence was substracted and results were normalized for protein content. To discard nonspecific effects of blebbistatin, DHE staining was measured in control and PoCo hearts reperfused for 5 minutes in the absence of blebbistatin (n=6 per group).

To determine the major source of ROS impacted by PoCo in our experimental model, additional groups of hearts subjected to the ischemia/reperfusion protocol with or without PoCo were perfused throughout the perfusion protocol with the xanthine oxidase inhibitor allopurinol (1 mmol/L, Sigma), the NADPH oxidase inhibitor apocinin (100 μmol/L, Sigma), or BH_4_ (100 μmol/L, Merck) (n=5 per group).

### Measurement of Nitrotyrosine Formation

The modification of protein tyrosine residues to 3‐nitrotyrosine, a footprint of in vivo ONOO^−^ formation, was measured by SDS‐PAGE Western blotting.^19^ Myocardial samples obtained after 5 minutes of reperfusion (n=3 per group) were prepared, and proteins were separated via electrophoresis on 12% SDS gels and transferred onto nitrocellulose membrane as described for protein phosphorylation. The primary 3‐nitrotyrosine antibody (Millipore) was used at 1:500. Samples from control and PoCo hearts perfused with the ONOO^−^ donor 3‐morpholinosydnonimine (SIN‐1 100 μmol/L, Tocris) added to the buffer during reperfusion were used as positive control.

### Measurement of Myocardial Biopterin

BH_4_ and BH_2_ contents were determined in cardiac homogenates obtained after 5 minutes of reperfusion (n=6). Hearts were rapidly excised and stored at −80°C. Samples were homogenized in buffer containing Tris‐HCl 50 mmol/L, EDTA 1 mmol/L, DTT 1 mmol/L, and 0.1 mg/ml ascorbic acid, pH 7.4 and centrifugated at 100 000*g* for 30 minutes. Concentration of biopterins were determined by high‐performance liquid chromatography analysis using a differential oxidation method as previously described^20^ and corrected for protein concentration. Control hearts supplemented with BH_4_ 100 μmol/L during reperfusion were used as positive control (n=5).

### Dimer:Monomer eNOS Ratio

Dimeric and monomeric forms of eNOS were analyzed in hearts reperfused for 5 minutes and homogenized in buffer containing Tris‐HCl 50 mmol/L, NaCl 150 mmol/L, EDTA 10 mmol/L, DTT 1 mmol/L, NaF 10 mmol/L, Na_3_VO_4_ 2 mmol/L, 1% Triton X‐100, and 1% Protease Inhibitor Cocktail (Sigma), pH 7.3 (n=3 per group). Proteins were separated via low‐temperature electrophoresis on a 6% SDS gel with Tris‐glycine‐SDS running buffer (Sigma), transferred in the presence of 0.01% SDS, and immunoblotted with antibody against total eNOS (BD Transduction Laboratories).^21^

### Nitrate/Nitrite Determination

Total nitrate/nitrite (NO_x_) levels were measured in myocardial and coronary effluent samples from control and PoCo groups obtained under normoxic conditions and at 3, 5, 10, and 30 minutes of reperfusion (n=5 for each time point) by using an NO_x_ fluorometric assay kit according to the manufacturer's directions (Cayman Chemical Co).^22^ Myocardial NO_x_ was also measured in control and PoCo hearts reperfused for 5 minutes with LY294002 (n=5), l‐NAME (n=5), BH_4_ (n=5), and SIN‐1 (n=4).To discard nonspecific effects of blebbistatin, myocardial NO_x_ levels were measured in control and PoCo hearts reperfused for 5 minutes in the absence of blebbistatin (n=5 per group).

### Measurement of Myocardial cGMP Content

Myocardial cGMP was measured in extracts^23^ from heart samples obtained from the different groups of treatment at 5 minutes of reperfusion (n=6 per group except groups reperfused with ODQ and l‐NAME [n=5 per group]) with a commercial cGMP EIA kit (Cayman Chemical Co) following manufacturer's instructions.

### Statistical Analysis

Data analysis was performed using SPSS for Windows. Means between groups were compared by 1‐way ANOVA or unpaired *t* test. Least significant difference test was applied as post‐hoc test when significant differences were observed. A 2‐way ANOVA was used to examine the effect of PoCo and the different drugs added at reperfusion on cell death and myocardial cGMP and NO_x_ content, DHE fluorescence, and the effect of PoCo and the time of reperfusion on protein phosphorylation and myocardial NO_x_. Significant differences between PoCo and its corresponding control group were determined by simple main effect analysis. Repeated‐measures ANOVA was used to compare temporal differences in LDH and NO_x_ released into the effluent. *P*<0.05 was considered to be statistically significant. The linear regression of protein phosphorylation on infarct size, corrected for group effect, was estimated using the general linear model with groups introduced as a fixed factor. In all cases, the assumption of normality was examined in SPSS before the statistical analysis using the Shapiro–Wilk test. When *P*>0.05, distribution of data was considered normal. All results are expressed as mean±SEM.

## Results

### NO‐Dependent Activation of cGMP/PKG Pathway, but Not PI3K/Akt Cascade, Mediates Postconditioning Protection

**Figure 2.**
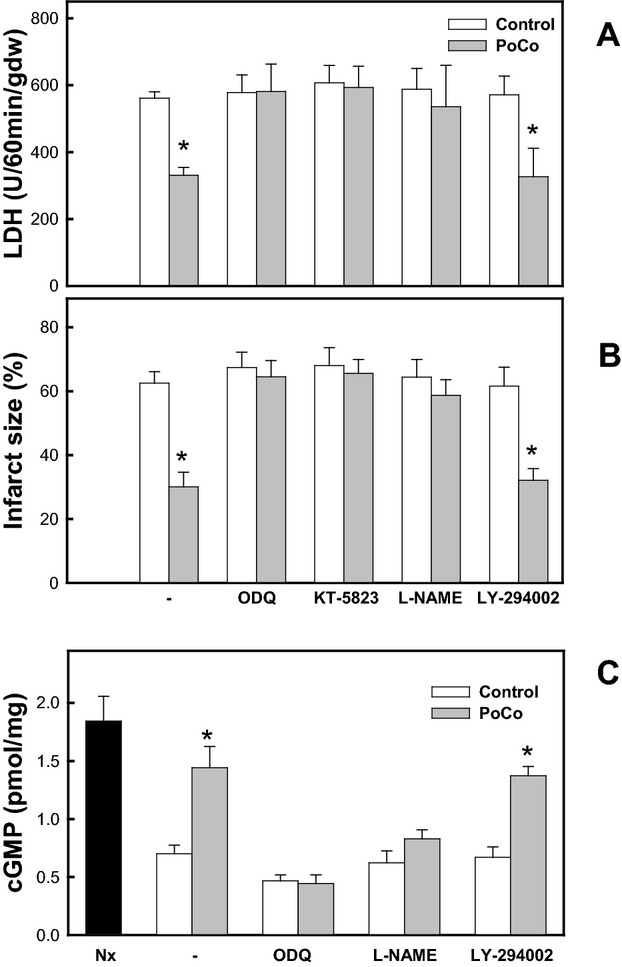
Quantification of reperfusion‐induced cell death and cGMP content in control and postconditioned (PoCo) hearts in the presence of the different inhibitors. A, Total LDH released during reperfusion and (B) infarct size measured by TTC and expressed as percentage of ventricular mass. C, Myocardial cGMP content measured in hearts perfused under normoxic conditions (Nx) and in hearts subjected to 40 minutes of ischemia and 5 minutes of reperfusion in the presence of blebbistatin. *P<0.05 vs control group. Data are mean±SEM. LDH indicates lactate dehydrogenase.

**Figure 3.**
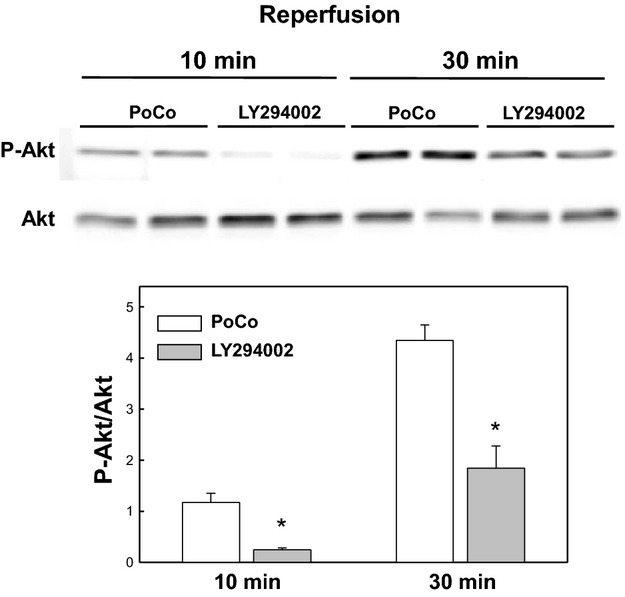
Effect of the phosphatidylinositol 3‐kinase (PI3K) inhibitor LY294002 on Akt phosphorylation measured after 10 and 30 minutes of reperfusion. *P<0.05 vs respective postconditioned (PoCo) group. Data are expressed as fold values compared with PoCo 10 minutes.

No differences among groups were observed in LV function during the equilibration and the ischemic period. At the end of the equilibration period, LVEDP and LV developed pressure were, respectively, 6.5±0.7 and 93.8±5.4 mm Hg, perfusion pressure was 63.1±3.7 mm Hg, and coronary flow was 12.3±1.5 mL/min. On the contrary, during the reperfusion period, hearts subjected to PoCo (PoCo group) showed delayed (3.8±0.2 versus 6.5±0.3 minutes in control group, *P*<0.001) and attenuated hypercontracture (138.2±7.9 versus 99.3±5.1 mm Hg, *P*<0.001) and markedly reduced LDH (*P*<0.001, Figure 2A) and infarct size (*P*<0.001, Figure 2B) and increased myocardial cGMP levels (*P*<0.001, Figure 2C). Treatment of hearts with either the soluble GC (sGC) inhibitor ODQ, the PKG blocker KT5823, or the NOS inhibitor l‐NAME abolished the effects of PoCo on hypercontracture, cell death, and cGMP content (Figure 2). In contrast, no effect of the PI3K inhibitor LY294002 on cell death and cGMP (Figure 2) was observed. Effective blockade of Akt phosphorylation by LY294002 administered during reperfusion was confirmed in heart samples obtained after 10 and 30 minutes of reperfusion, demonstrating that the lack of effect on cell death obtained with PI3K/Akt blockade was not the consequence of inadequate drug administration (Figure 3).

### Phosphorylation of RISK Is a Consequence but Not a Cause of Postconditioning Protection

**Figure 4.**
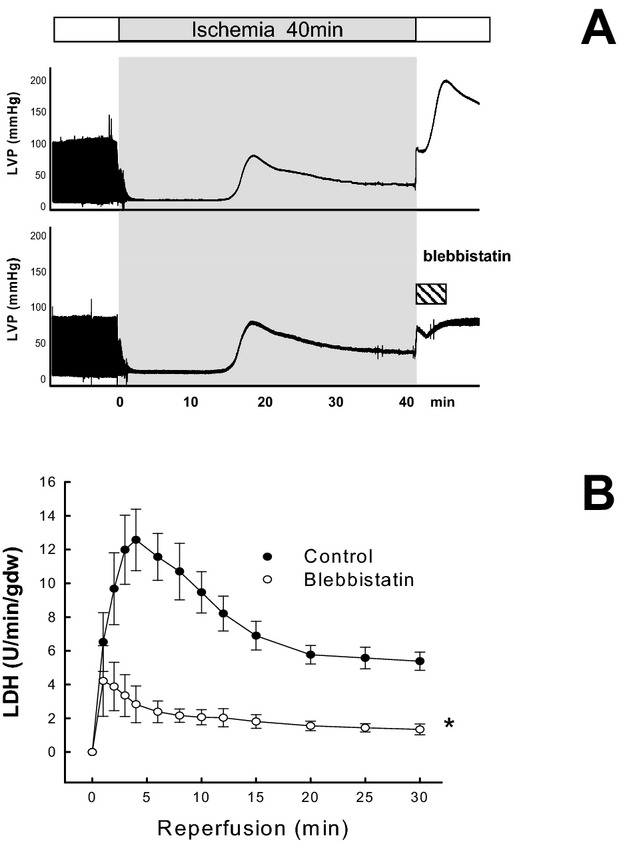
Effect of blebbistatin on left ventricular pressure (LVP) and cell death. A, Representative LVP corresponding to control (upper panel) and hearts receiving blebbistatin during 5 minutes of reperfusion (lower panel). B, Time course of LDH release during the first 30 minutes of reperfusion. *P<0.05. Data are mean±SEM. LDH indicates lactate dehydrogenase.

**Figure 5.**
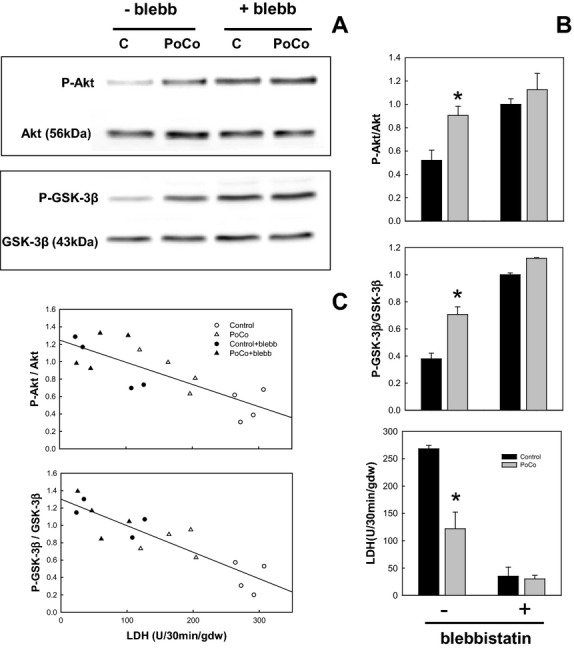
Correlation between phosphorylation of phosphatidylinositol 3‐kinase (PI3K)/Akt pathway and cell death. A, Western blot showing the phosphorylation of Akt and GSK‐3β in control and postconditioned (PoCo) hearts after 30 minutes of reperfusion with and without blebbistatin. B, Quantification of phosphorylation and LDH released during 30 minutes of reperfusion in the different groups. C, Scatter plot and regression analysis showing the correlation between the magnitude of Akt and GSK‐3β phosphorylation and cell death (LDH). *P<0.05 vs respective control group. Data are mean±SEM. LDH indicates lactate dehydrogenase.

The addition of the contractile inhibitor blebbistatin to the perfusion buffer at the onset of reperfusion prevents the development of hypercontracture (Figure 4A) and cell death associated with mechanical stress (Figure 4B). Blebbistatin was used to discard the possibility that any variation on protein phosphorylation was a consequence of differences in cell death. The use of blebbistatin abolished the differences between the control and PoCo groups in the extent of cell death (Figure 5).

PoCo hearts reperfused for 30 minutes showed a marked increase in the phosphorylation of Akt (*P*<0.001) and in the RISK downstream effector GSK‐3β (*P*<0.001) with respect to control hearts. However, when blebbistatin was added during reperfusion, and cell death in both control and PoCo hearts was markedly reduced, differences between groups in protein phosphorylation measured at 30 minutes of reperfusion were abolished (Figure 5A and 5B). Blebbistatin had no effect on protein phosphorylation in normoxically perfused hearts, demonstrating that the increased phosphorylation observed during reperfusion is a consequence of reduced cell death and not of a blebbistatin‐dependent activation of RISK. A scatter plot that included control and PoCo groups reperfused with and without blebbistatin disclosed a close correlation between infarct size and phosphorylation. For Akt, the general linear model analysis shows a significantly negative regression of protein phosphorylation on infarct size (*P*<0.039), the effects of groups, corrected for covariation with infarct size, being nonsignificant (*P*<0.282). A similar pattern was observed for GSK‐3β but, in this instance, the test for negative regression did not reach significance (*P*<0.085). It is worth noting that the regression slope computed from the pooled data by ignoring groups is significantly negative in both instances (*P*<0.0022 for Akt, *P*<0.0021 for GSK‐3β) (Figure 5C).

**Figure 6.**
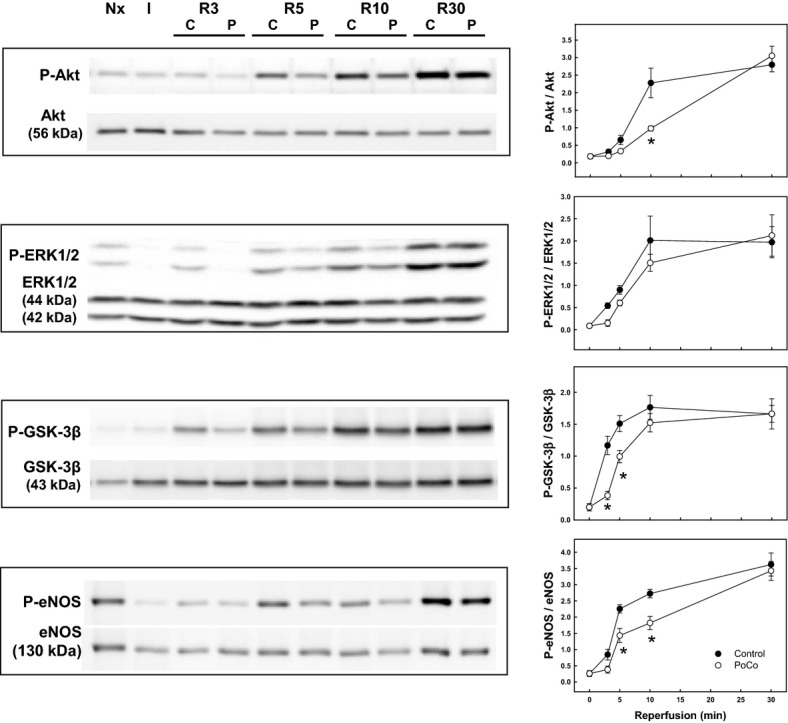
Time course of Akt, ERK1/2, GSK‐3β, and eNOS phosphorylation in samples from hearts corresponding to control (C) and postconditioned (P) groups obtained before reperfusion and after 3, 5, 10, and 30 minutes of reperfusion in presence of blebbistatin (R). Data are expressed as fold increase compared with ischemia (I). Nx corresponds to hearts normoxically perfused for 60 minutes. *P<0.05 vs respective control group. eNOS indicates endothelial nitric oxide synthase.

The time course of protein phosphorylation in control hearts treated with blebbistatin showed a sustained increase in phosphorylation of all measured proteins during reperfusion with a plateau at 10 minutes for ERK1/2 and GSK‐3β and at 30 minutes for Akt and eNOS (Figure 6). PoCo produced a delay in protein phosphorylation of Akt/GSK‐3β during the first minutes of reperfusion, although the initial differences were progressively reduced, and after 30 minutes of reperfusion, phosphorylation of all measured proteins was similar between control and PoCo groups (Figure 6).

### An Alternative Mechanism: Postconditioning Increases NOS Coupling by Preserving BH_4_

**Figure 7.**
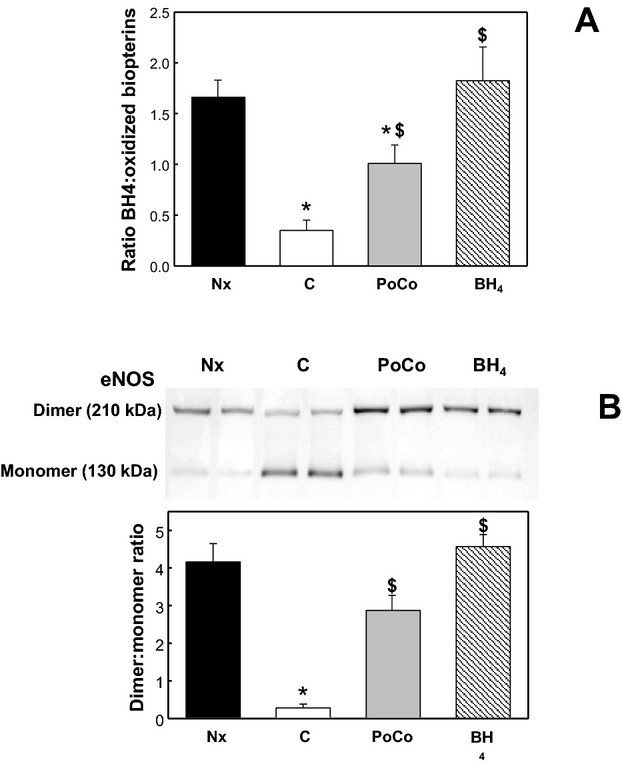
Effect of postconditioning on BH_4_ and eNOS coupling after 40 minutes of ischemia and 5 minutes of reperfusion. A, Quantification of the ratio between reduced and oxidated BH_4_. B, Representative Western blot and quantification of the SDS‐resistant eNOS dimer. Positive control was obtained by adding external BH_4_ to the buffer during reperfusion. *P<0.05 vs Nx group. ^$^*P*<0.05 vs control group. Data are mean±SEM. Nx indicates normoxically perfused group; C, control group; PoCo, postconditioned group; eNOS, endothelial nitric oxide synthase; BH_4_, tetrahydrobiopterin.

Myocardial BH_4_ concentration measured in control hearts was markedly depleted after 5 minutes of reperfusion (1.02±0.22 versus 3.08±0.37 pmol/mg protein in normoxically perfused hearts, *P*<0.001). These values are similar to those reported previously in the isolated rat heart.^12^ PoCo increased the ratio of reduced BH_4_ to oxidized biopterins, although without reaching preischemic values of BH_4_ (*P*=0.038 compared with control hearts) (Figure 7A).

To investigate the effect of BH_4_ preservation on the stability of eNOS dimer, the ratio between dimeric and monomeric forms of eNOS was determined through separation of heart homogenates using low‐temperature electrophoresis. The results obtained demonstrated a close correlation between the reduced:oxidized biopterins ratio and the eNOS dimer:monomer ratio. The dimeric form of eNOS was markedly reduced in homogenates from control hearts measured after 5 minutes of reperfusion, whereas PoCo significantly increased the dimeric and reduced the monomeric eNOS (*P*<0.001). Perfusion with external BH_4_ produced an additional increase in the dimer:monomer ratio (Figure 7B).

**Figure 8.**
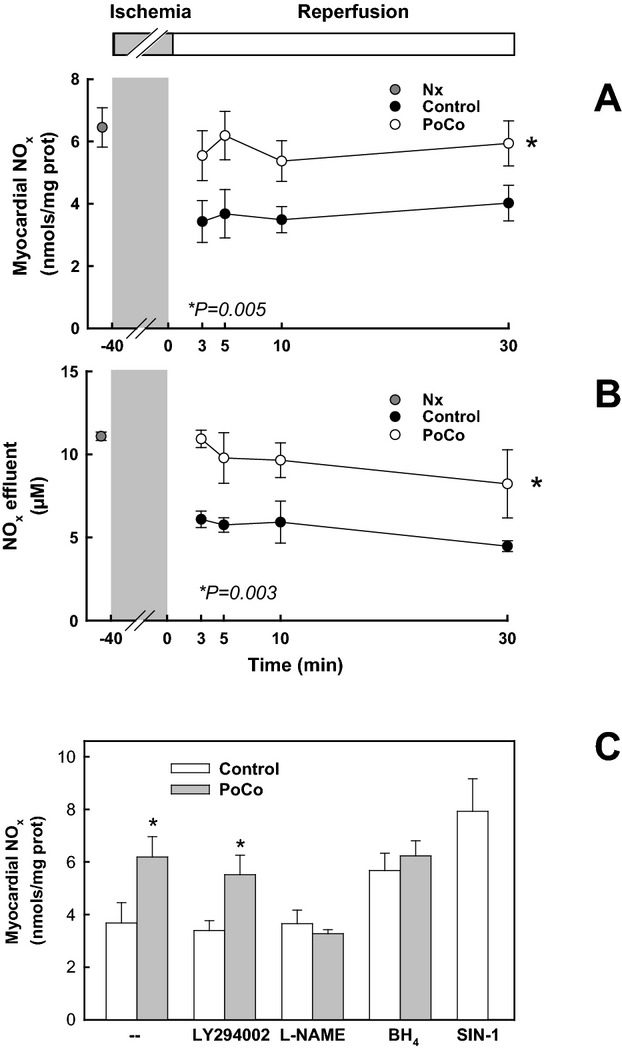
Effect of postconditioning on nitrate/nitrite concentration (NO_x_). A, Myocardial NO_x_ content and (B) NO_x_ released to the coronary efluent in normoxically perfused hearts (Nx) and at 3, 5, 10, and 30 minutes of reperfusion after 40 minutes of ischemia in control and preconditioned (PoCo) hearts. C, Myocardial NO_x_ content measured in heart samples obtained after 40 minutes of ischemia and 5 minutes of reperfusion. *P<0.05 vs respective control group. ^$^*P*<0.05 vs untreated control group.

The increased eNOS coupling in PoCo hearts was confirmed by measuring the time course of NO_x_ generated in myocardium and released into the coronary effluent during the first 30 minutes of reperfusion. NO_x_ levels fell in myocardium at the onset of reperfusion in control hearts and showed no significant recovery in myocardium (Figure 8A) and a gradual decrease in the coronary effluent (Figure 8B). PoCo attenuated NO_x_ drop at the onset of reperfusion and levels remained higher than in control hearts throughout the reperfusion period (*P*=0.005 in myocardial samples and *P*=0.003 in samples from coronary effluent). As shown in Figure 8C, perfusion with l‐NAME but not with LY294002 attenuated the production of NO_x_ observed in PoCo myocardial samples obtained after 5 minutes of reperfusion. BH_4_ increased NO_x_ level in the control group (*P*=0.032 compared with untreated control group) and had no additive effect when combined with PoCo. Increased NO_x_ production in PoCo hearts was confirmed in an additional group of hearts perfused without blebbistatin (3.13±0.22 nmol/mg protein in control hearts versus 5.58±0.206 nmol/mg protein in PoCo hearts, *P*=0.009).

### Postconditioning Preserves BH_4_ by Reducing O_2_^−^ Production and Oxidative Stress

**Figure 9.**
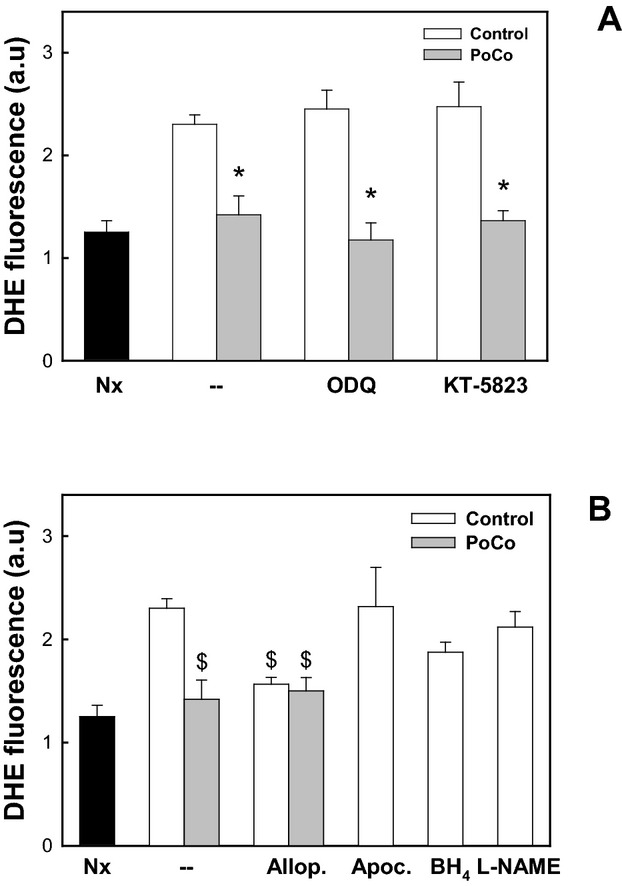
Measurement of O_2_^−^ production using DHE after 40 minutes of ischemia and 5 minutes of reperfusion. A, Effect of ischemic postconditioning and the cGMP/protein kinase G) pathway on the production of O_2_^−^. B, Effect of inhibitors of ROS generation. Hearts were perfused with the xanthine oxidase inhibitor allopurinol (Allop.), NADPH oxidase inhibitor apocynin (Apoc.), or the reactive oxygen species cofactor BH_4_. DHE fluorescence is expressed as arbitrary units (a.u). *P<0.05 vs Nx group. ^$^*P*<0.05 vs control group without treatment. Data are mean±SEM. Nx indicates normoxically perfused group; C, control group; PoCo, postconditioned group; ODQ, 1*H*‐[1,2,4]oxadiazolo[4,3‐**a**]quinoxalin‐1‐one; DHE, dihydroethidium; BH_4_, tetrahydrobiopterin; O_2_^−^, superoxide.

Superoxide production was assessed by DHE oxidation fluorescence. Fluorescence measured after 5 minutes of reperfusion with DHE in the presence of blebbistatin increased in control hearts compared with normoxically perfused hearts (*P*<0.001, Figure 9A) and was prevented by PoCo. Inhibition of the cGMP/PKG pathway with ODQ or KT‐5823 did not modify fluorescence at the onset of reperfusion, confirming that O_2_^−^ reduction by PoCo is upstream PKG activation. Prevention of O_2_^−^ production by PoCo was confirmed in an additional group of hearts perfused without blebbistatin (2.58±0.09 AU in control hearts versus 1.83±0.11 AU in PoCo hearts, *P*=0.001).

The addition of allopurinol to the perfusion buffer reduced DHE fluorescence in control hearts (*P*<0.001 versus untreated control group), whereas perfusion with apocynin or l‐NAME had no effect. The perfusion of hearts with external BH_4_ produced a nonstatistically significant trend toward attenuation (*P*=0.116 versus control group). None of these treatments had an additive effect when combined with PoCo. These results suggest that the major pool of O_2_^−^ reduced by PoCo is generated by xanthine oxidase (Figure 9B).

**Figure 10.**
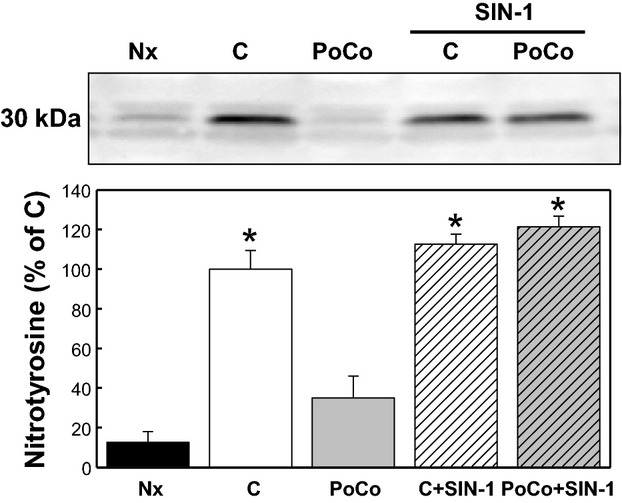
Effect of ischemic postconditioning on the production of ONOO^−^ after 40 minutes of ischemia and 5 minutes of reperfusion. Representative Western blot showing a nitrotyrosylated 30‐kDa band, a marker of endogenous ONOO^−^ generation. Positive control was obtained by perfusing the hearts with the ONOO^−^ donor 3‐morpholinosydnonimine (SIN‐1). *P<0.05 vs Nx group. Data are mean±SEM. Nx indicates normoxically perfused group; C, control group; PoCo, postconditioned group; ONOO^−^, peroxynitrite.

To confirm that PoCo reduces O_2_^−^ and attenuates the oxidative stress at the onset of reperfusion, protein nitrotyrosilation was measured by Western blotting as an index of in vivo ONOO^−^ formation. In samples corresponding to control hearts reperfused for 5 minutes immunoblotted against 3‐nitrotyrosine, a marked band of ≈30 kDa appeared that was significantly attenuated in the PoCo group (*P*<0.001, Figure 10). The specificity of this band was confirmed by perfusing the hearts with the ONOO^−^ donor SIN‐1. Perfusion of PoCo hearts with SIN‐1 increased the formation of nitrotyrosine to values similar to those obtained in control hearts.

Administration of BH_4_ during the first minutes of reperfusion produced a nonstatistically significant trend toward infarct size reduction (61.9±4.8% in control group versus 51.3±3.5% in postconditioned group, *P*=0.111) and resulted in increased protein nitrosylation.

## Discussion

The present study demonstrates that the increased NO‐dependent cGMP/PKG signaling induced by PoCo is due to reduced formation of O_2_^−^ at the onset of reperfusion resulting in attenuated oxidation of BH_4_, which reduces NOS uncoupling and increases NO availability. Our results further support an important role of cGMP in PoCo protection but suggest that the increased phosphorylation of RISK observed in PoCo hearts is a consequence but not a cause of the reduction in cell death. These results should assist in the development of therapeutic strategies aimed to reduce reperfusion injury and infarct size in patients.

Myocardial cGMP/PKG signaling is severely depressed in cardiomyocytes and endothelial cells after prolonged ischemia.^23–24^ There is abundant evidence supporting that preservation of this pathway with NO donors, natriuretic peptides, or cGMP analogs attenuates cell death associated with reperfusion.^23,25–26^ More recently, the blockade of cGMP/PKG pathway by using NOS, sGC, or PKG inhibitors abolished the infarct‐sparing effects of PoCo, demonstrating that cGMP‐mediated signaling is involved in the mechanisms responsible for PoCo‐induced cardioprotection.^6–7^ Preservation of cGMP by PoCo has been suggested to participate in different molecular mechanisms through the activation of PKG. PKG has been shown to inhibit the opening of mitochondrial KATP,^27^ attenuate Ca^2+^ oscillations,^26^ and reduce the sensitivity of myofibrils to Ca^2+^.^23,28^ More recently, it has been proposed that PKG activation contributes to PoCo protection by delaying normalization of pHi during reperfusion, probably via inhibition of Na‐H exchanger.^7^

PKG is commonly admitted to be a downstream effector of the PI3K/Akt cascade.^29^ According to this pathway, phosphorylation of the RISK component Akt results in phosphorylation‐dependent activation of eNOS and the consequent NO‐dependent activation of sGC. However, recent studies in different models suggest that RISK phosphorylation during reperfusion is not associated with a reduction in infarct size.^9,30–31^ By comparing protein phosphorylation in myocardial biopsy samples obtained from viable regions within the area at risk after reperfusion with and without PoCo, it has been demonstrated that RISK phosphorylation is not causally involved in ischemic PoCo in pigs.^9^ Our data go beyond these studies in that they provide a plausible explanation for the discrepancies in the role of RISK. Studies showing an association of RISK phosphorylation with less infarction used animal models in which the small amount of viable tissue makes technically impossible the approach taken by Skyschally et al in pigs and, therefore, the samples collected from the area at risk contains more viable cells in PoCo myocardium resulting in an increased phosphorylation potential. This technical limitation was also tested by Skyschally et al in comparing protein phosphorylation from necrotic and viable myocardium samples obtained after 10 minutes of reperfusion, and they found no differences.^9^ Although authors suggested that tissue udergoing infarction was not dead yet, they did not measure phosphorylation after more prolonged reperfusion. In our study, to rule out the possibility that the effects of PoCo on protein phosphorylation were just a reflection of its effect on cell death, hearts were reperfused in the presence of the contractile inhibitor blebbistatin. Contractile inhibition has been consistently demonstrated to attenuate mechanical stress associated with hypercontracture and to prevent sarcolemmal rupture and cell death at the onset of reperfusion.^14,32–33^ Using this approach, we demonstrated a close correlation between the magnitude of protein phosphorylation and the extent of cell death that is independent of the perfusion protocol. In the absence of differences in cell death, phosphorylation of Akt, ERK, and their proposed downstream effectors eNOS and GSK‐3β was similar in both groups after 30 minutes of reperfusion or even lower in the PoCo group during the first 10 minutes, a time interval of critical importance in reperfusion injury.^34^ Moreover, blockade of NOS, sGC, and PKG, but not PI3K, abolished the cardioprotective effects of PoCo, confirming that activation of the cGMP/PKG pathway depends on activation of NOS but cannot be explained by the activation of PI3K/Akt cascade.

In the present study, we propose an alternative mechanism to explain the NO‐dependent activation of cGMP/PKG pathway by PoCo. Numerous studies describe a burst of ROS within the first minutes of reperfusion and support their contribution to reperfusion injury.^35^ It has been suggested using an in vivo rat model that PoCo may reduce ROS generation immediately at reperfusion by limiting the delivery of oxygen during the controlled reperfusion.^36^ Our results confirm that PoCo attenuates the formation of O_2_^−^ at the onset of reperfusion, suggest that this reduction mainly involves O_2_^−^ produced by xanthine oxidase activity, and demonstrate that the associated reduction of oxidative stress mediates the eNOS‐dependent activation of the cGMP/PKG pathway. The lack of effect of sGC or PKG inhibition on O_2_^−^ production supports that ROS reduction by PoCo is upstream PKG activation. Attenuation of O_2_^−^ generation by PoCo may have different effects. O_2_^−^ combines with NO at a very fast rate to form ONOO^−^, a potent oxidant implicated in a number of pathophysiological processes.^37^ Peroxynitrite not only reduces the availability of free NO but also, due to its high reactivity and diffusibility,^38^ produces toxic effects through the oxidation of lipids, sulfhydrils, DNA, and redox molecules and the nitration of protein tyrosine residues.^39^ Prevention of ONOO^−^ formation during reperfusion has been generally associated with reduced reperfusion injury.^40–41^ In the present study, by measuring protein nitrotyrosilation, a fingerprint of the presence of ONOO^−^ in vivo, and using as positive control samples from hearts perfused with the ONOO^−^ donor SIN‐1, we demonstrate that PoCo attenuates the formation of ONOO^−^. These results are consistent with a recent study showing a reduction of nitro‐oxidative stress by PoCo in an in vivo rat model.^42^ In that study, the reduction in protein nitrosylation by PoCo was interpreted as a consequence of inhibited iNOS activity. However, no expression of iNOS after 5 minutes of reperfusion has been observed in our experimental model.

ONOO^−^ may act directly on the eNOS heme group, producing the inactivation of the enzyme.^43^ In addition, O_2_^−^ and/or ONOO^−^ can oxidize BH_4_, a cofactor essential for proper function of NOS,^44^ leading to NOS uncoupling and preferential production of O_2_^−^ rather than NO.^45–46^ BH_4_ is dramatically reduced during ischemia–reperfusion,^12^ and administration of BH_4_ has been found to ameliorate NOS activity and to reduce reperfusion injury.^12,47^ Our results confirm the oxidation of BH_4_ at the onset of reperfusion and demonstrate that PoCo attenuates BH_4_ depletion and increases eNOS coupling as evaluated by the increase in the dimeric form of eNOS and in the nitrite levels in the coronary effluent. The BH_4_:oxidated biopterins ratio closely correlated with the ratio of dimeric to monomeric forms of eNOS. NOS uncoupling has been shown to increase O_2_^−^ generation in postischemic hearts.^12^ In our study, BH_4_ supplementation was associated to a nonstatistically significant trend toward reduced O_2_^−^ production, suggesting that O_2_^−^ generated by uncoupled eNOS was not the main cause of the reduction of oxidative stress by PoCo, and failed to reduce infarct size. The failure of BH_4_ to protect against reperfusion injury despite increasing eNOS coupling and NO_x_ levels can be explained according to the mechanism proposed in Figure 8. Although PoCo preserves BH_4_ by reducing ROS production, the administration of external BH_4_ during reperfusion increased the levels of BH_4_ without significantly reducing myocardial ROS. In agreement with our data, it has been recently shown that oral BH_4_ treatment augments the levels of biopterins in patients with coronary artery disease without affecting vascular function or O_2_^−^ production.^48^ In our study, perfusion with BH_4_ increased the formation of peroxynitrite, an effect that could counterbalance the potential cardioprotective actions of BH_4_.

Besides the activation of the cGMP/PKG pathway, other signaling events have been proposed to be decisive in PoCo protection that are compatible with the results obtained in our study. PKG activation contributes together with reduced lactate washout to delay pHi correction,^7^ which has a beneficial effect on many mechanisms implicated in reperfusion injury,^49^ including attenuation of the mechanical stress associated to development of hypercontracture and formation of mitochondrial permeability transition. It can be hypothesized that by preventing these critical events during early reperfusion, prolongation of acidosis allows the activation of the survivor activating factor enhancement (SAFE) pathway to continue inhibiting mitochondrial permeability transition after pHi normalization. In fact, the activation of mitochondrial signal transducer and activator of transcription 3 (STAT‐3) has been recently proposed to mediate postconditioning cardioprotection through better mitochondrial function.^50^ Furthermore, besides activation of PKG, ROS scavenging is a means of reducing a major stimulus for mitochondrial permeability transition during reperfusion.^51–52^

A potential limitation of our study is the use of an isolated rodent heart. The perfusion with a cristalloid buffer, which requires higher oxygen tension and lacks of the intrinsic erythrocyte antioxidant defenses, may exaggerate the production of ROS. However, it has been proposed that in the Langendorff model, O_2_ tension is inversely related to the burst of radical production^53^ and that oxygen free radicals are generated in even higher concentration at reperfusion after regional ischemia in situ than after global ischemia in the isolated perfused rabbit heart.^54^ However, there is agreement that there are differences in the time course of ROS generation between isolated heart and the in situ models. Spin trap resonance shows a maximal free radical intensity within the first minute of reperfusion before quickly dropping to near baseline levels in the isolated heart model,^55^ whereas in the in situ model, after the first burst of ROS, neutrophil infiltration prolongs O_2_^−^ generation mainly through NADPH oxidase activity.^56^ Because PoCo mediates its effects during the first minutes of reperfusion, which are of critical importance for reperfusion injury, our results may still be valid in the in vivo model.

Our results suggest that PoCo attenuates O_2_^−^ produced by xanthine oxidase activity. In earlier studies, xanthine oxidase has been identified as an important source of ROS at reperfusion in rodents.^57^ However, its contribution as a source of O_2_^−^ generated during ischemia–reperfusion in the human heart remains controversial. Although some studies suggest that xanthine oxidase is not expressed in human myocardium,^58^ other studies demonstrate its presence and the reduction of markers of oxidative stress in patients treated with allopurinol.^59–61^ Because the burst of ROS generated at the onset of reperfusion has been described in humans and protocols that limit the delivery of oxygen, such as PoCo, reduce ROS generation independently of their source, we are convinced that our results have translational relevance.

**Figure 11.**
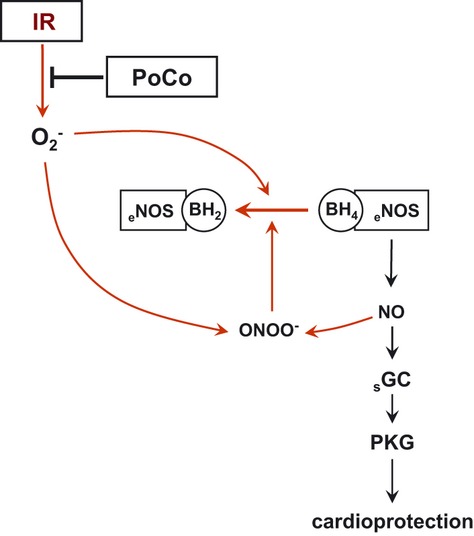
Proposed mechanism for NO‐dependent activation of cGMP/protein kinase G (PKG) pathway by postconditioning (PoCo). The present study provides data (in red) demonstrating that PoCo attenuates the burst of O_2_^−^ generated at the onset of reperfusion, reducing oxidative stress and eNOS uncoupling. NO indicates nitric oxide; ONOO^−^, peroxynitrite; O_2_^−^, superoxide; eNOS, endothelial nitric oxide synthase; BH_4_, tetrahydrobiopterin; sGC, soluble guanylyl cyclase.

In conclusion, our data demonstrate that activation of the PKG/cGMP pathway by PoCo in the isolated and perfused rat heart is independent of PI3K/Akt activation and dependent on reduced oxidative stress during reperfusion. Our study shows that attenuation of O_2_^−^ and/or ONOO^−^ at the onset of reperfusion by PoCo limits the oxidation of BH_4_ and reduces eNOS uncoupling, increasing NO‐dependent activation of cGMP/PKG pathway (Figure 11). These results contribute to the better understanding of the mechanisms underlying ischemic PoCo, a critical step necessary for its translation to the clinical scenario, and should orientate the development of pharmacological interventions able to reproduce the cardioprotective effects of PoCo in patients subjected to reperfusion after myocardial ischemia.
